# Molecular Communication Between Neuronal Networks and Intestinal Epithelial Cells in Gut Inflammation and Parkinson's Disease

**DOI:** 10.3389/fmed.2021.655123

**Published:** 2021-07-22

**Authors:** Alice Drobny, Phuong A. Ngo, Markus F. Neurath, Friederike Zunke, Rocío López-Posadas

**Affiliations:** ^1^Department of Molecular Neurology, University Hospital Erlangen, Friedrich-Alexander-University Erlangen-Nuremberg, Erlangen, Germany; ^2^Medicine 1, University Hospital Erlangen, Erlangen, Germany; ^3^Deutsches Zentrum Immuntherapie, Erlangen, Germany

**Keywords:** Parkinson's disease, gut-brain axis, enteroendocrine cells, alpha-synuclein, intestinal inflammation, inflammatory bowel diseases

## Abstract

Intestinal symptoms, such as nausea, vomiting, and constipation, are common in Parkinson's disease patients. These clinical signs normally appear years before the diagnosis of the neurodegenerative disease, preceding the occurrence of motor manifestations. Moreover, it is postulated that Parkinson's disease might originate in the gut, due to a response against the intestinal microbiota leading to alterations in alpha-synuclein in the intestinal autonomic nervous system. Transmission of this protein to the central nervous system is mediated potentially via the vagus nerve. Thus, deposition of aggregated alpha-synuclein in the gastrointestinal tract has been suggested as a potential prodromal diagnostic marker for Parkinson's disease. Interestingly, hallmarks of chronic intestinal inflammation in inflammatory bowel disease, such as dysbiosis and increased intestinal permeability, are also observed in Parkinson's disease patients. Additionally, alpha-synuclein accumulations were detected in the gut of Crohn's disease patients. Despite a solid association between neurodegenerative diseases and gut inflammation, it is not clear whether intestinal alterations represent cause or consequence of neuroinflammation in the central nervous system. In this review, we summarize the bidirectional communication between the brain and the gut in the context of Parkinson's disease and intestinal dysfunction/inflammation as present in inflammatory bowel disease. Further, we focus on the contribution of intestinal epithelium, the communication between intestinal epithelial cells, microbiota, immune and neuronal cells, as well as mechanisms causing alterations of epithelial integrity.

## Introduction

### Parkinson's Disease

Parkinson's disease (PD) is the second most common neurological disorder characterized by movement disabilities (1), but also by non-motor symptoms, including gastrointestinal dysfunction that often appears years before diagnosis of disease (2, 3). A neuropathological hallmark of PD is the aggregation of the synaptic protein alpha-synuclein (aSyn) within the central nervous system (CNS), leading to degeneration of dopaminergic neurons within the *substantia nigra pars compacta* (SNpc) of the midbrain (1). Moreover, research suggests that inflammatory responses within the CNS contribute to PD pathology. Hence, glial cell reactions and T cell infiltration result in increased levels of inflammatory cytokines within the CNS and are currently recognized as prominent features of PD (4, 5).

### Intestinal Dysfunction and Inflammation Within PD

Interestingly, recent data indicate that intestinal inflammation contributes to the pathogenesis of PD (6), and increasing numbers of studies imply that PD may start in the gastrointestinal system years before any motor symptoms develop (7–9). An acute and chronic intestinal inflammation is a prominent feature of Inflammatory bowel disease (IBD) comprising the diseases Ulcerative colitis (UC) and Crohn's disease (CD). While UC mainly affects the colon and rectum, CD injures the entire GI tract (10). IBD is understood to be a result of gut microbiota dysbiosis and mucosal immune dysregulation (11). Also, intestinal inflammation in IBD is associated with intestinal epithelial cell (IEC) alterations and maintaining epithelial homeostasis helps in protecting against inflammation (12). Remarkably, PD and IBD share overlapping genetic factors found within a recent genome-wide-association study (GWAS) (13). The leucin-rich repeat kinase 2 (*LRRK2*) gene appears to be the most susceptibility-factor for both diseases (14, 15). Interestingly, *LRRK2* is one of the genes most commonly associated with familial and sporadic PD (16). Recent studies show, that patients with IBD have a higher risk of developing PD as compared to non-IBD individuals (17, 18). It is well-established that IBD is characterized by chronic pro-inflammatory immune activity (11), which is now suggested to be a fundamental element of neurodegenerative disorders as well (5, 6). Furthermore, animal studies demonstrate that gut inflammation, similar to IBD, induces loss of dopaminergic neurons (19, 20). Additionally, chronic GI inflammation is likely to induce anxiety-like behavior and alter CNS biochemistry in mice (21). Interestingly, CD patients have been shown to accumulate aSyn in the gut (22).

Moreover, aSyn and its aggregated forms were also found in the enteric nervous system (ENS) of PD patients and symptoms outside the CNS were described including GI impairments (2, 23, 24). This gave rise to the hypothesis that PD pathology can spread from the gut to the brain and vice versa (23, 25, 26). This hypothesis is supported by recent animal studies, which recapitulated the transmission of aSyn pathology via the vagal nerve, connecting the central with the peripheral nervous system (27, 28). In this context, the discovery of aSyn expression in enteroendocrine cells (EECs) within the intestinal epithelium suggests these cells as sensors of luminal signals triggering the gut-neural circuit behind aSyn alteration (29, 30). This signal is then transmitted to the CNS, potentially via the vagus nerve. Thus, deposition of aggregated aSyn in the GI tract has been inferred as a potential diagnostic marker for prodromal PD.

**Figure 1:**
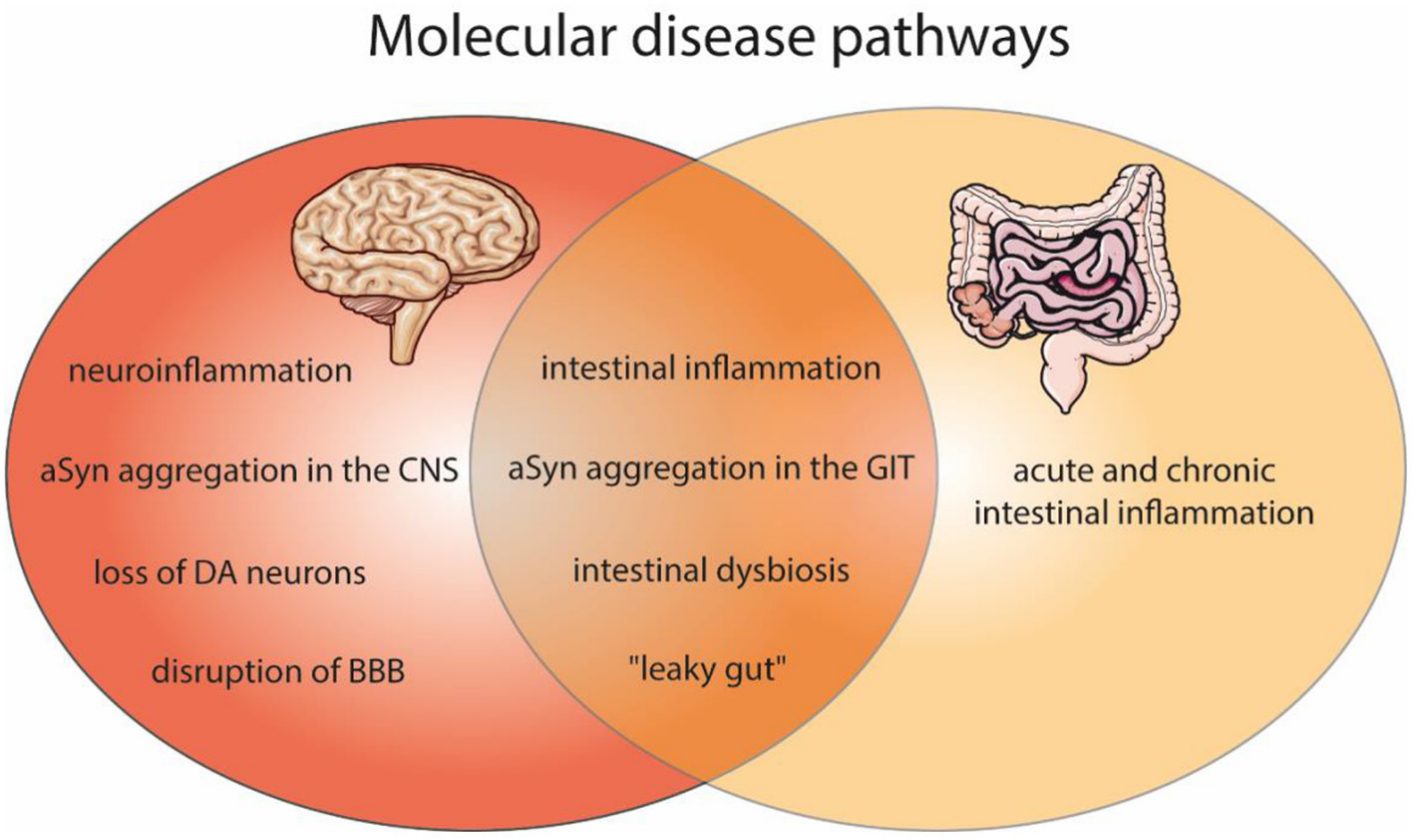
Shared molecular disease pathways of the brain and gut pathologies, as found in PD and IBD. Affected molecular features within PD (red) and gut inflammation (light orange) as well as in both disease (dark orange). Molecular pathways of PD include neuroinflammation, aSyn aggregation in the central nervous system (CNS), dopaminergic neurons (DA) degeneration, and the disruption of blood-brain barrier (BBB). In IBD an acute and chronic intestinal inflammation is described. Both diseases can comprise intestinal inflammation, aSyn aggregation in the gastrointestinal tract (GIT), intestinal dysbiosis and a “leaky gut.” The figure contains modified components of Servier Medical Art, licensed under the Creative Commons Attribution 3.0 Unported License (CC BY 3.0) https://smart.servier.com.

**Figure 2:**
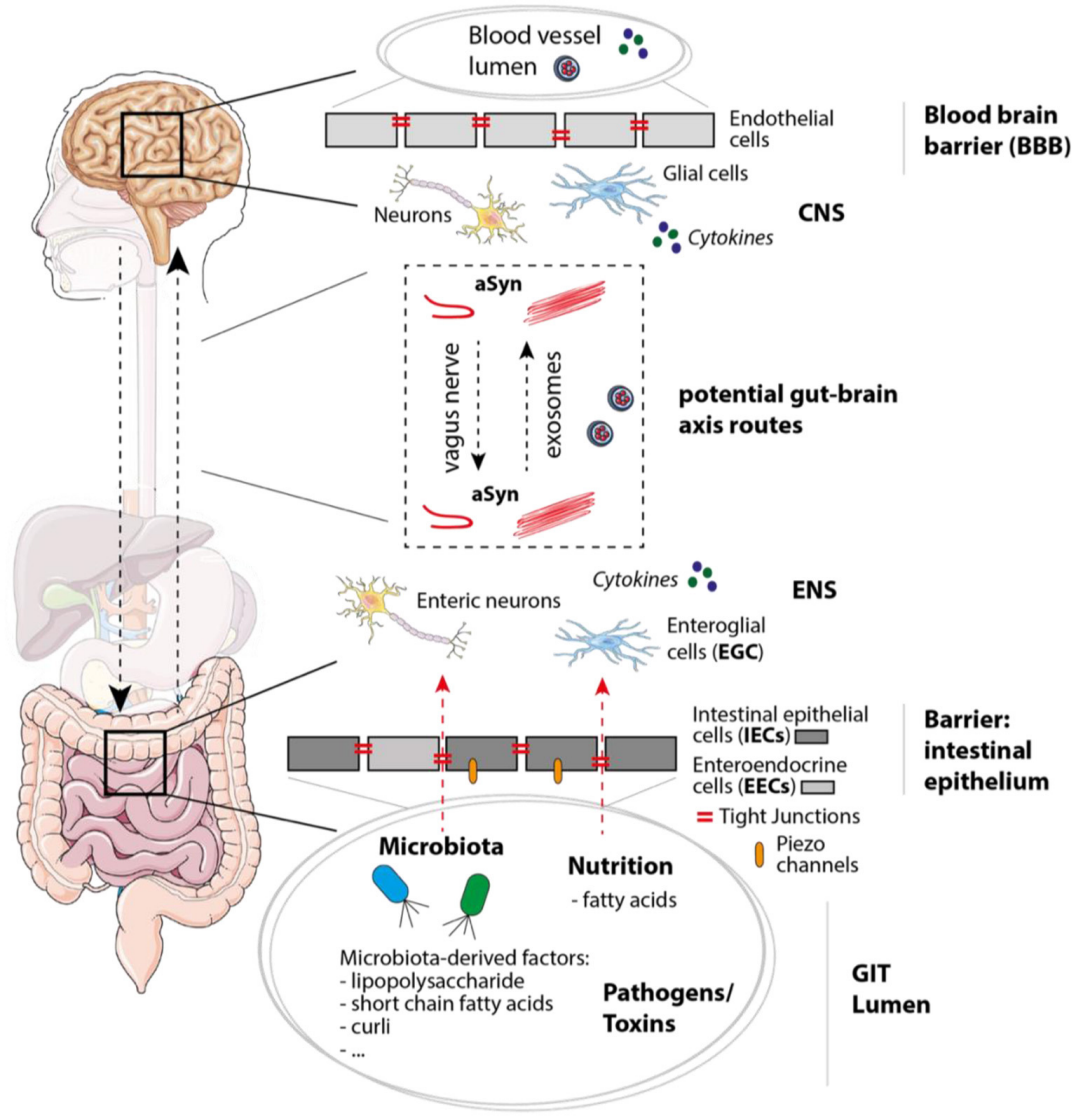
Mechanisms, molecules, and cell types involved in PD/IBD pathology and gut-brain communication. In the gastrointestinal tract (GIT) the intestinal epithelium functions as barrier and separates the GIT lumen from the surrounding enteric nervous system (ENS), which not only contains enteric neurons, but also enteroglial cells. Within the GIT lumen microbiota and microbiota-derived factors like polysaccharides, short chain fatty acids, and curli (bacterial amyloid protein) can be found, but also nutritions, possible pathogens and toxins. When integrity of the intestinal epithelium consisting of epithelial cells including enteroendocrine cells (IECs and EECs) is disturbed, molecules within the GIT lumen get in contact with cells of the ENS. Tight junctions and Piezo channels have been shown to play an important role in mechanosensation, peristalsis, and intestinal barrier function. Within the brain, the blood-brain barrier (BBB) separates the CNS (including neurons and glial cells) from the blood vessel lumen. Potential gut-brain axis routes on which aSyn and molecules like cytokines could be transferred are for instance the vagus nerve or exosomes [via the blood stream or cerebrospinal fluid (CSF)]. The figure contains modified components of Servier Medical Art, licensed under the Creative Commons Attribution 3.0 Unported License (CC BY 3.0) https://smart.servier.com.

This review focuses on overlapping disease pathologies and the molecular communication between the brain and the gut in the context of PD and gut inflammation, as present in IBD (Figure 1). We emphasis on the contribution of neurodegeneration and neuroinflammation in PD, gut-brain spreading of PD pathology, intestinal epithelium and the communication between IECs, microbiota and immune cells (Figure 2). Moreover, we discuss the mechanisms causing alterations of epithelial integrity and gastrointestinal (GI) dysfunction in PD.

## Neuropathology in PD

### Motor and Non-motor Manifestations of PD

PD is clinically characterized by classical motor symptoms including muscular rigidity, bradykinesia, rest tremor, and postural instability (1). Among several putative factors that may contribute to PD pathology, the most crucial indication of PD is the degeneration of neurons in the CNS. The loss of dopaminergic neurons within the SNpc is the most predominant feature during disease progression (31) and leads to excessive dopamine depletion within the basal ganglia, which results in the above mentioned parkinsonian motor characteristics (1). The administration of the amino acid precursor of dopamine, L-DOPA (L-3,4-dihydroxy-L-phenylalanine), has shown to be the most effective symptomatic treatment. However, if the motor symptoms occur in PD patients the continuous loss of neurons is already inexorable (32). Interestingly, PD manifests already >20 years before the motoric problems occur. This premotor or prodromal period of disease is defined by e.g., constipation, olfactory dysfunction, sleep disorder, cognitive impairment, autonomic dysfunction, pain and fatigue (1, 3). Altogether, this leads to the assumption that PD is a complex, multisystem disorder with both neurologic and systemic non-motor manifestations.

### Lewy Body Pathology in the CNS

A neuropathological hallmark of PD is the formation of intracellular amyloid inclusions in neuronal bodies and neurites, known as Lewy bodies (LB) and Lewy neuritis (LN), respectively, consisting of aggregated aSyn (33). The appearance of these aSyn-carrying inclusions in patients is also collectively known as synucleinopathies, referring to PD, dementia with Lewy bodies (DLB) and multiple system atrophy (MSA) (33–35).

Physiologically, aSyn is natively unfolded and soluble with an amphipathic N-terminus, a hydrophobic central domain known as non-amyloid-β component (NAC) region, and an acidic C-terminus (36). Under pathological conditions, aSyn aggregates have been shown to exert cell toxic properties (37, 38). The aggregation mechanisms, by which soluble aSyn changes its structure to oligomers and ultimately to insoluble β-sheet rich fibrils, are still under debate (39–41). However, several factors have been described to induce structural changes of monomeric aSyn, involving the interactions with specific lipids (42, 43) and membranes (44). Further, posttranslational modifications such as phosphorylation (45), nitration and oxidation (46), ubiquitination (47), and sumoylation (48) have been shown to accelerate aSyn pathology. Multiple copies (e.g., duplications and triplications) (49, 50) and missense mutations (e.g., A53T, A30P, E46K, and H50Q) (51–53) of the gene encoding for aSyn (*SNCA*) foster protein aggregation. In addition, the cellular microenvironment of aSyn has been reported to play a role in aSyn conformation and solubility. For instance, aSyn aggregation behavior differs between neutral pH7.4 (e.g., cytosol) and acidic pH5 (e.g., lysosome) and aSyn purified from lysosomes was able to seed aggregation in a concentration-dependent manner (54, 55). Interestingly, dysfunction in lysosomal pathways have been linked to PD (56, 57). For degradation, aSyn is processed within lysosomes by specialized lysosomal enzymes (cathepsins) (58–60). Hence, deficiency within these lysosomal enzymes, important for lysosomal function and aSyn degradation, lead to its aggregation and pathology (61–64). Targeting lysosomal enzymes by boosting their activity has become a promising therapeutic approach, which might lower aSyn burden within neuronal cells and thus decrease the risk of pathological aSyn aggregation and neurotoxicity (65, 66). In a nutshell, intracellular accumulation of aSyn, due to inefficient clearance mechanisms, might drive further aggregation of the protein (67). In this regard, it was shown that toxic aSyn species can be released to the periphery from stressed and/or dying neurons and are subsequently taken up by surrounding cells, leading to spreading of pathology (68–70). Especially, aggregation intermediates, such as aSyn oligomers, exhibit highly cell toxic properties (43, 71, 72).

### Neuroinflammation in PD

In recent years, evidence evolved that aSyn and inflammatory processes are extraordinarily connected. In that sense, chronic neuroinflammation is another characteristic indicator of PD pathophysiology and is considered to promote the progression of dopaminergic cell death (73, 74). In general, neuroinflammation is defined as the immune response of cells within the brain and plays an important role in maintenance of nervous tissue homeostasis (4). On the one hand, a moderate inflammation can protect neurons from damage (75); on the other hand, inflammatory factors do also affect neurons directly and convey neurodegeneration. In addition, neuronal cell death induces inflammatory mechanisms, and contributes to a vicious cycle of inflammation and progressive loss of neurons in the brain (76). The neuroinflammatory response is mediated by resident immune cells (microglia and astrocytes), which release cytokines and chemokines (4, 77).

Many neuroinflammatory circumstances at *post-mortem* stage have also been identified on a molecular basis in PD. For example, numerous proinflammatory cytokines and factors such as tumor necrosis factor (TNF)-α, β2-microglobulin, epidermal growth factor (EGF), transforming growth factor α (TGFα), TGFβ1, and interleukin (IL)-1β, IL-6, and IL-2 were found in the striatum of PD patients (78). Furthermore, TNF-α, IL-1β, and interferon (IF)-γ were also detected in the SNpc of PD patients (79). Interestingly, dopaminergic neurons express the receptors of these cytokines (80), that might explain the vulnerability of DA neurons to inflammatory processes inside the brain. In addition, increased levels of proinflammatory mediators, such as IL-1β, IL-2, TNF-α, and IL-6 are present in the serum and the cerebrospinal fluid (CSF) of PD patients (81–84). These results suggest the direct migration of immune cells from the periphery (blood stream) to the brain (or vice versa) during neurodegenerative process.

Microglia, the resident macrophages in the brain, and astrocytes, the most abundant glial subtype in the CNS, are considered to drive the inflammatory response in PD (85). Of relevance, microglia initiate the innate immune response in the brain, therefore representing key players upon inflammatory stimulus (86, 87). Under pathological conditions, activated microglia release proinflammatory cytokines and reactive oxygen species (ROS), which affect dopaminergic neuron viability (73, 88). Reactive microglia were found in various brain regions (89, 90) including the SNpc of PD patients (91). Besides microglia activation, reactive astrogliosis contributes to PD pathogenesis and progression (85). Astroglial cells secret the glial cell-line derived neurotrophic factor (GDNF), which promotes survival of dopaminergic neurons (92), and regulates the permeability of the blood-brain-barrier (BBB) (93, 94). Interestingly, in this regard the BBB is found to be defective in PD patients (95–97). The mechanism of an altered BBB function is still elusive, however, the increased levels of proinflammatory cytokines IL-6, IL-1β, and TNF-α have been associated with a disruption of trans-endothelial electrical resistance, indicating an increased BBB permeability (98). Recently, a study showed that aSyn-mediated release of proinflammatory cytokines and chemokines by pericytes induces disruption of BBB (99). Further, accumulations of aSyn in astrocytes are found in *post-mortem* analysis of PD patients (100). Reactive astrocytes manifest with PD progression by increased proinflammatory cytokine secretion such as IL-1β, TNF-α, and IFN-γ (101–103). A recent study indicates the close interplay between microglia and astrocytes showing induction of neurotoxic A1 astrocytes by microglial secretion of IL-1α, TNF-α and complement component 1q (C1q) (104). In this regard, it was shown that pathological aSyn inoculation *in vitro* and *in vivo* induces microglia to secrete cytokines and chemokines followed by astrocyte A1 activation that caused neuronal cell death in culture and neurodegeneration in mice (105).

Moreover, is has been reported that aSyn itself has an important role in the initiation and maintenance of inflammation in PD. Recent reports have suggested that aSyn acts as a damage-associated molecular pattern (DAMP), capable of modulating inflammatory cytokine production in microglia and inducing intracellular signaling cascades (106, 107). It has been demonstrated that extracellular oligomeric aSyn is a putative activator of toll-like receptor (TLR) 2 and promotes microglia-mediated inflammatory cytokine and ROS production (108, 109). The exact contribution of different aSyn conformations to TLR activation is currently unclear, however, there are strong indications that the activation seems to be conformation dependent. Specifically, TLR4 appears to be involved in the uptake of fibrillary aSyn (110). While the presence of monomeric aSyn seems to enhance phagocytic function, aggregated forms seem to inhibit this process (111).

Furthermore, lymphocyte infiltration might also play a role in inflammation processes inside the brain of PD patients. It was reported that cytotoxic T lymphocytes (CD8+) as well as CD4+ T helper (Th) cells were more abundant in the brains of PD patients compared to healthy individuals (112). In this regard, T cells, in particular Th17 cells, were increased in number in PD brain and blood. Furthermore, Th17 cells induced cell death in co-cultures autologous induced pluripotent stem cells (iPSC)-derived neurons from PD patients (113). Interestingly, aSyn is able to activate helper and cytotoxic T cell responses in PD patients, which suggests a possible role of autoimmune inflammation in PD (114).

Taken together, these current data implicate that PD is an extraordinary complex disease with many pathophysiological processes driving disease progression. It becomes evident, that PD is rather a systemic disorder with a variety of pathological facets than ‘just' neurological degeneration.

## Gut-Brain Axis in PD

Approximately 80% of PD patients suffer from GI manifestations (115), including constipation, which seems to be an important risk factor for PD (116). As mentioned above, intestinal symptoms may precede motor manifestations by several years, suggesting that PD might originate in the gut. This is in line with the estimation that 90% of idiopathic PD cases are due to oral ingestion of substances causing cell toxicity (oxidative stress, mitochondrial dysfunction), such as herbicides and pesticides (117). According to the hypothesis that PD originates from the gut (118), aggregates of aSyn were detected in the intestine of PD samples (119). Despite the evidence of gut to brain communication in the context of PD, there are still open questions regarding (A) the exact localization in the gut where PD might originate, (B) the dissemination pathways within the gut and to the brain, (C) the declutching event of proteinopathy in the gut, and (D) the role of the intestinal microbiota, as well as microbiota-epithelial-immune communication. All this lacking information is indispensable in order to develop potential PD diagnosis strategies based on GI premotor symptoms.

### aSyn in the Gut and Its Propagation to the CNS

As already mentioned, LBs and different aSyn conformers were observed in variety of organs despite the brain. aSyn was reported to be present in the spinal cord and the peripheral nervous system (PNS) including the paravertebral sympathetic ganglia, vagus nerve, the GI tract and among others (120, 121). Indeed, phosphorylated aSyn, a pathological form of aSyn, has been detected in the GI tract up to 20 years before onset of PD motor symptoms (9). Also, Braak and colleagues hypothesized that synucleinopathy begins in the anterior olfactory nucleus and the dorsal motor nucleus of the vagus nerve (DMV) (dual-hit theory) (8, 23), favoring the idea that PD pathology invades the brain via retrograde axonal transport (25, 26). Braak and colleagues even suggest that a pathogen, a pathogen-derived component or other exposures are entering the nervous system through axons of the myenteric (Auerbach's) plexus and/or the submucosal (Meissner's) plexus via postganglionic neurons and may trigger aSyn conformation to aggregates and fibrils (8, 122). Thus, the microbiota has been suggested as a key player, since local immune activation can lead to systemic inflammation affecting the BBB, finally causing neuroinflammation and neurodegeneration (123) (Figure 2). Although it is still not clear whether microbiota changes are cause or consequence, dysbiosis is considered as a risk factors for PD development.

#### Detection of aSyn in the Gastrointestinal Tract

In the context of the ENS, aSyn was first identified in the esophagus and the colon (124), but it is still not clear where the deposition under pathological conditions initiates. Current literature demonstrates that aSyn can also be detected in salivary glands (125), pharyngeal sensory nerves (126), the esophagus (120), the stomach and the small intestine (127), the colon (123), and the appendix (128). Colonic aSyn has been detected even in premotor PD (119, 129). These observations postulate detection of intestinal aSyn as a diagnostic tool in PD, even in early phases of the disease. However, inconsistencies in the detection of aSyn conformers imply the need of alternative and more accurate methods for its detection (granular staining in the lamina propia, perivascular/vascular wall mucosa staining, lacy-granular pattern in the submucosa, or epithelial cell nuclear staining, 2D/3D electrophoresis) (24).

Monomeric aSyn expressed in gut neurons can be released in form of free protein or exosomes, which can be taken up by neighboring neurons via endocytosis (130). Most commonly, aSyn is transported directly from neuron to neuron (131, 132), which requires close cellular contacts and intact synaptic connections (133). In the gut, this is possible via the connection between submucosal/myenteric neurons to the preganglional vagal nerves, which allows aSyn propagation (134). Proteinopathy within the GI innervation might be due to a neurotropic pathogen/agent, which initiates Lewy pathology in the gut (8). Therefore, a connection between the ENS and the mentioned agent is necessary, since neurons/nerves do not reach the intestinal lumen. An attractive candidate in this context would be the intestinal epithelium, which is in direct contact with luminal content, and therefore acts as a physical and immunological barrier in the gut. On the other hand, disturbances of intestinal sealing in chronic intestinal inflammation leading to leaky gut might allow direct contact of the initiating factor and the ENS (Figure 2) (135–137).

#### Propagation of aSyn Between the Gut and the CNS

The next important question is how aSyn propagates from the ENS to the CNS. The connection between the ENS and the CNS, so called gut-brain axis, permits a mutual effect from the ENS to the CNS, and vice versa. This communication mainly occurs via the sympathetic system and the vagus nerve of the autonomic nervous system, and the spinal cord. Four levels of control have been defined (138): (1) ENS, including myenteric and submucosal ganglia, and enteroglial cells; (2) prevertebral ganglia (visceral reflex responses); (3) spinal tract the through tractus solitaires in the brain stem and the dorsal motor nucleus of the vagus nerve; and (4) cortical and basal ganglia neurons. Healthy individuals maintain intestinal functions, and patients with neurodegenerative disease suffer from GI problems, not only PD, but also Alzheimer's disease, transmissible spongiform encephalopathies, or amyotrophic lateral sclerosis; while GI disorders leads to CNS-related symptoms. The connection between the vagus nerve and the luminal content has been suggested to be mediated via EECs (139), which might produce metabolites acting on the vagus nerve, transmitting information from the nutrients toward the brain, in a glutamatergic neurotransmission (140) (Figure 2).

The vagus nerve, one of the largest nerves connecting the gut and brain, is considered to be the direct link between these two organs (141). Recent data from rodent models could evaluate a direct propagation of aSyn pathology from the gut to the brain via the vagal nerve (28, 142–144) (see section Animal Models of PD and GI Symptoms). Moreover, there has also been also research in alternative hypothesis of a brain-to-gut spread of aSyn pathology, showing that a vector-mediated overexpression of aSyn in the midbrain lead to accumulations of aSyn in enteric nerves and stomach walls (145). Further, a more recent study presents that a nigral overexpression of aSyn exerts significant alteration on the ENS followed by changes in the microbiome (146). Subsequently, loss of neuronal plexus and activation of glial cells in the gut impact on intestinal permeability, barrier function, inflammation, and GI motor functions. Taken together, this data suggests a bidirectional potential of aSyn to move both anterogradely and retrogradely within neurons (Figure 2). If the vagus nerve is the main route of bidirectional aSyn transmission, vagotomy could be protective against developing PD. Studies questioning whether a vagotomy leads to a reduced risk to develop PD could not find a strong association (147, 148). Only when the cases of a full truncal vagatomy were restricted >20 years after surgery a decreased risk for subsequent PD was observed (149). Overall, many studies support the idea of aSyn gut to brain and inversely brain to gut spread, however, there are still clinical studies missing that investigate the start and/or early development of PD progression, respectively.

Another way of aSyn transmission from the gut to the brain and vice-versa is thought to be possible through extracellular vesicles called exosomes, which are found in the blood serum and CSF of PD patients (Figure 2) (150–152). In fact, it was shown that exosomes derived from PD patients incorporate oligomeric aSyn and spread oligomerization of aSyn in a dose-dependent manner (130, 153, 154). An alternative gut-brain communication via the circulation has been also suggested in primates, where the damage of the CNS could be observed upon intestinal injection of aSyn without affecting the vagus nerve, but elevated aSyn levels in the circulation (155). Overall, this indicates that exosomes may function as intracellular cargo distributing aSyn pathology throughout the body (Figure 2).

### Enteric Nervous System (ENS)

Being the largest and most complex part of the peripheral nervous system (PNS), the ENS controls crucial functions within the gastrointestinal tract, such as peristalsis, substance transport, or local blood supplies. The ENS innervates the whole GI tract, from the mouth to the rectum, including the salivary glands. Neuron networks in the gut wall formed ganglia, which are interconnected by dense fiber bundles. The nerve plexuses are organized in myenteric and submucosal plexuses, which are, in turn, interconnected. The myenteric plexus is localized between the longitudinal and circular muscle layers throughout the GI tract, and controls smooth muscle activity and motility. The submucosal plexus is located mainly in the small and large intestine, also in the stomach, but not in the esophagus (156).

ENS-mediated control of the GI function is independent from the CNS; therefore, the ENS allows complete sensory-motor reflexes, based on the existence of primary afferent neurons, interneurons and motor neurons. However, apart from this intrinsic innervation within the ENS, the gut is also innervated via the sympathetic and parasympathetic nervous system. More than 100 million entities from 20 different neuron subtypes (depending on the expression of neuropeptides) coexist with enteroglial cells (EGCs) in the ENS. EGCs express glial fibrillary acidic protein (GFAP), vimentin and S-100, but also receptors for cytokines, neuropeptides and neurotrophins, and therefore, have a dual function on the ENS. As astrocyte-like cells they also contribute to the function of the intestinal immune system. Moreover, ECGs participate in the structure of the ENS and contribute to the maintenance of mucosal barrier and tissue homeostasis (157). Interestingly, EGCs also serve as a communication tool between IECs and the ENS (158). Among ENS neurons, dopaminergic neurons are present in both plexus (159), and are more frequent in the proximal part of the GI tract; although the association between the loss of dopaminergic neurons and PD has been only demonstrated in the colon (160).

### Microbiota

Seeing it as a super-organism, the human body is not only composed of human cells but also numerous microorganisms colonizing at mucosal surfaces, allowing various important body's functions such as maturation, education of host immune responses, protection against pathogen proliferation, and induction of responses to specific drugs. The human gut-microbiome carries millions of microorganisms and indeed, has been defined as the most complex ecosystem ever. It contributes greatly to intestinal immune function as a consequence of the continuous contact with gut lumen commensals and potentially harmful agents. A symbiotic relationship between the human body and these microorganisms permits the digestion of nutrients and pathogen colonization resistance. Thus, the intestinal microbiota modulates several functions of the gastrointestinal tract, such as permeability (161), mucosal immune function, motility (162), sensory nerve function and ENS activity (163). Interestingly, it is also associated with brain functions (164), such as response to stress (165), emotions (138), pain, digestive behavior (166), and brain biochemistry (167).

The balance between the human body and the microbiota (eubiosis) is challenged by several external factors, such as antibiotic treatment, various diseases, highly processed foods or lack of sleep. This can lead to microbiota alterations or dysbiosis, which in most cases is shown by variations in the composition and reduced diversity between different species. Dysbiosis has been associated with several pathological situations, including IBD (168) and PD (169) (Figure 1); although in many cases it is not clear whether its alteration represents cause or consequence of the subjacent pathology. The most accepted hypothesis for the pathogenesis of IBD claims that chronic intestinal inflammation occurs as an exacerbated immune response against components of the microbiota in genetically predisposed individuals. The first hint pointing to the association between the intestinal microbiota and IBD came from animal studies showing that experimental inflammation in a number of well-established animal models was abolished in germ-free mice (170). In addition, inflammation could be challenged upon colonization with caecal bacteria, while specific species were able to protect upon recolonization. Despite numerous efforts in order to identify a single specie capable of triggering chronic intestinal inflammation (171), nowadays IBD is considered as a polymicrobial disease, where dysregulation in the composition of the microbiota affects several species. In addition to activation of signals upon detection of the microbiota or derived-antigens, another important aspect is the release of metabolites derived from the microbiota. This has been identified using next-generation sequencing, metagenomics and metabolomics, allowing the description of the microbiome and its potential alterations (172, 173).

Based on the relevance of the intestinal microbiota, its modulation in order to restore eubiosis, appears as an attractive strategy for therapy purposes. In this context, fecal transplantation implies the transfer of microbiota from healthy donors to IBD patients. Fecal microbiota transplantation (FMT) has been tested in various pathological conditions, such as IBD, diabetes type 2 and even neurodegenerative disease. A recent study demonstrates the efficacy of this strategy in an experimental colitis model induced by adoptive transfer of naïve T cells, since transfer of healthy vs. IBD patient fecal content permits restoration of T cell responses (decreased Th17/Th2); and increased Treg/IFNγ and ameliorates thereby colitis (174). Despite limitations based on the donor testing, the limited duration of the treatment and the potential alterations upon antibiotic treatment, FMT it is approved for the treatment of other intestinal conditions, such as *Clostridium difficile* infections (175, 176). In addition to fecal transplantation, a recent review collects other therapy strategies based on the modulation of the microbiota via direct or indirect mechanisms, such as enteral nutrition; pre-, pro-, and post-biotics; inhibition of Adherent-invasive *Escherichia coli* (AIEC) adhesion and tungstate treatment (168). All these strategies to restore eubiosis are potentially valuable in diverse pathologies coursing with dysbiosis.

#### Microbiota in PD

Compared to GI homeostasis, more surprising is the association between microbiota and brain function, and the fact that the intestinal flora modulates immune, endocrine, and neuroendocrine maturation in nervous system sprouting. Colonization of the human gut upon birth is important for neonatal brain development, since it allows the synthesis of vitamins and fatty acids, regulation of BDNF (Brain-derived neurotrophic factor), synaptophysin and PSD-95 (177). Experimentally, sterile mice elicit decreased expression of BDNF in the cerebral cortex and hippocampus, and they show signs of anxiety and less activity performance (178); while another study shows that recolonization with healthy flora permitted production of different neurotransmitters (NTs) and the abolition of anxiety symptoms (179). An additional important aspect to be considered is the ability of the microbiota to directly produce inhibitory NT (GABA) or regulate their synthesis by the host (180, 181). Moreover, GABA signaling system (GAD and GABA_A_R) was detected in IECs and GABA_A_R stimulation played important role in regulating intestinal fluid secretion in rat (182). On the other hand, preventing the reuptake of NTs (for example, inhibiting 5-HT reuptake by fluoxetine) can regulate colonization in the gut (183). In addition, important to mention here is the production of short-chain fatty acids (SCFA) as microbiota-derived factors, which can affect the CNS thank to their passaging through the BBB via specific transporters. SCFAs in the brain regulated microglia homeostasis (184), have impact on G-protein coupled receptors (GPCRs) (185, 186) and maintain to the GPR41-mediated SNS activity (187). According to an association between brain function/development and colonization of the intestinal tract, the microbiota impacts then on social behavior, sleep cycle, mood disorders, and neurodegenerative disease including Alzheimer's disease and PD (188).

In the context of the gut-brain axis, components of the microbiota and its metabolites can act directly on neurons at the ENS, or signal through IECs (Figure 2) (189). Nowadays, several pieces of evidence demonstrate a correlation between dysbiosis and prodromal signs in PD (190–192). Importantly, changes affecting *Firmicutes, Prevotella, Helicobater pylori* (193), *Bacteroides*, or *Bifidobacterium* (194) as well as the imbalance between pro- and anti-inflammatory species, and the increased release of LPS should be mentioned (195). Based on a recent Metabolome wide association studies (MWAS) (196), the dysbiosis in PD patients is characterized by: increase of opportunistic pathogens (*Porphyromonas, Corynebacterium, Prevotella, Porphyromonas*, and *Corynebacterium*); reduction of SCFA-producing bacteria (*Oscillospira, Lachnospiraceae*_UCG-04, *Lachnospiraceae*_ND3007_group, *Agathobacter, Butyricicoccus, Blautia, Faecalibacterium, Lachnospira, Fusicatenibacter, Roseburia*); and elevated carbohydrate-metabolizing probiotics becoming immunogenic (*Lactobacillus* or *Biffidoacerium*). An independent meta-analysis of 223 PD vs. 137 control patients from America and Europe suggests elevation of *Akkermansia, Catabacter genera*, and *Akkermansiaceae* family together with reduction of general *Roseburia* and *Faecalibacterium* (197). Beyond alterations of the microbiota composition, related metabolic changes have also been observed in PD patients, such as reduced carbohydrates fermentation, butyrate synthesis, increased proteolytic fermentation, and amino acid metabolism (198). Interestingly, some of these metabolites play crucial roles for nervous system-related intestinal functions; for instance, SCFA contribute to 5-HT release and colon motility, proving again the gut-brain connection (199).

Interestingly, many of the PD-related microbiota alterations can also be linked to dysbiosis in IBD. *Akkermansia muciniphila* is a well-known actor in the context of IBD, since it can degrade the mucus layer and thereby impair the barrier function (200), which might favor the contact between the luminal content and the ENS. On the other hand, *Roseburia* and *Faecalibacterium* (197) possess an anti-inflammatory effect in IBD, due to their ability to produce SCFAs (201, 202); while decreased *Prevotellaceae* is associated with alterations of intestinal permeability via a similar mechanism (203). On its part, accumulation of *Enterobacteriaceae* leads to increased levels of LPS, explaining its correlation with disease progression and motor symptoms. Increased LPS levels can contribute to GI alterations by several mechanisms, such as causing epithelial leakage (204), inducing the production of cytokines and inflammation. Moreover, it can pass through the BBB (205), triggering direct destruction of the substantia nigra (206). Based on the neuroprotective effect of SCFA and ghrelin, reduced *Lactobacillaceae* can also affect intestinal inflammation, correlating with disease severity (207). Jointly, overlaps between dysbiosis profiles in IBD and PD might contribute to the associated barrier function alterations.

Changes in the gut microbiota composition might lead to aSyn accumulation in the gut, originating oxidative stress and mucosal inflammation. However, it is not clear whether changes in the microbiota composition, PD associated symptoms (constipation) or PD pharmacological treatment are a consequence of aSyn proteinopathy. Supporting a causative role of microbiota and/or microbiota-derived factors, a recent study shows induction of motor symptoms in mice upon fecal transplantation from human PD patients, due to aSyn pathology and neuroinflammation engendered by microbiota metabolites, such as SCFA. Furthermore, aSyn overexpressing mice (under the Thy1-promoter) show less motor symptoms in germ-free conditions, as well as upon antibiotic-treatment; while colonization with healthy or, in particular, PD patient-derived microbiota, lead to worsening of motor symptoms (192).

Beyond commensal bacteria, also pathogens in the lumen interact with the ENS, mostly via non-neuronal cells, such as EECs within the intestinal epithelium. On the other hand, local gut infections can impact on affective state and emotional responsiveness. This communication occurs via toxins promoting secretion and therefore, diarrhea, as observed in the case of *Vibrio cholera, Clostriiodes difficile*; or toxins promoting emesis, including *Staphyloccoccus aureus* or *Bacillus cereus*. However, not only bacteria, also viruses and parasites demonstrate an interplay with the ENS and CNS. The viremic hit hypothesis defends that PD occurs upon Influenza and HSV1 infections (dual-hit theory), leading to the aSyn aggregation in peripheral nervous tissues, and subsequently propagation to the brain (208–210). Interestingly, HIV targets the ENS, since it activates glial cells, which can then be propagated to the CNS. Furthermore, HIV Tat peptide can synergize with LPS by interfering with TLR4, inducing the release of cytokines, and promoting the proinflamamtory effect of LPS (211). ENS infection by HSV-1 leads to macrophage recruitment, releasing ROS and causing ENS neuroplasticity and destruction of enteric ganglia as well as GI dysmotility (212). Finally, parasites modulate 5-HT secretion, the release of enzymes degrading NTs, such as acetylcholinesterases (*Anisakis* or *Schistosome*), and NT secretion, while they are tightly linked to the immune system function (213).

### Intestinal Epithelium and “Leaky Gut”

The intestine is in charge of nutrition and water/ion absorption, but represents also a fundamental immunological organ, harboring the most extended immune cell population in the body. On its part, the intestinal epithelium together with the attached mucus constitute a physical and immunological barrier segregating the environment (intestinal lumen) and the human body. The gut epithelium consists of a monolayer of columnar epithelial cells allowing trans- and para-cellular transport required for nutrition, however, simultaneously impairing the invasion of potentially harmful pathogens. Thus, sealing of the epithelium has to be tightly maintained, in order to prevent transmucosal passage of microbiota-derived factors, which can then get in contact with the plethora of immune cells present in the sub-epithelial space. This is achieved via intercellular junctions (tight junctions, adherens junctions, and desmosomes) (214), as well as a tight regulation of cell architecture and polarity, mostly regulated by the function of the actin-myosin cytoskeleton (12, 215). Together, the intestinal epithelium accounts for the intestinal barrier function, which has been critically involved in the pathogenesis of intestinal disorders, such as chronic intestinal inflammation (including IBD) (216). This barrier function is challenged during the renewal or turnover of the epithelial layer. Lgr5^+^ stem cells located at the crypt bottom proliferate and give rise to pluripotent daughter cells located in the transient-amplifying area, which, in turn, differentiate into five IEC subtypes [enterocytes, goblet cells, paneth cells, EECs, and tuft cells (217)]. All differentiated IECs, except paneth cells which remain at the crypt, migrate upwards to the villus tip (small intestine) or the crypt surface (colon), where aged cells will be extruded to the lumen (cell shedding) and finally die. Temporary leakage occurring at the villus tip is tightly regulated by rearrangement of tight junctions and the so-called zipper effect of neighboring cells (218, 219), which allows resealing of the epithelium.

As mentioned above, the maintenance of epithelial integrity plays a fundamental role to keep tissue homeostasis in the gut, and therefore, avoid inflammation (220). Loss of epithelial sealing and leakage of the intestinal layer has been associated with chronic inflammatory disorders, such as IBD (221). Indeed, some observations claim that epithelial-intrinsic alterations can play a causative role in the disease. For instance, increased intestinal permeability appears in non-diagnosed relatives of IBD patients (222), and precedes flares in patients with an IBD diagnosis (223), suggesting that epithelial leakage heads the activation of the inflammatory response. Moreover, based on immune-epithelial communication in the gut, epithelial architecture and function can also be modified due to the effect of pro-inflammatory mediators present in the gut mucosa upon activation of an immune response, such as immune-cell derived cytokines (TNF, IL-6, IL-1β, IL-13, etc.). These cytokines affect mainly tight junction assembly (224), activation of different cell death pathways or cell shedding (225), as well as IEC damage (226). Altogether, via epithelial intrinsic and extrinsic mechanisms, epithelial barrier function is challenged in the context of IBD, and this correlates with pathogenesis of the disease. A proof of this association are recently introduced epithelial restoration therapy strategies, which indeed show promising results in the context of IBD pharmacological management (227, 228).

Beyond being a pure physical fence against components present in the lumen, the intestinal epithelium displays innate immune responses based on the expression of pattern-recognition receptors (PRRs), allowing them to recognize pathogen-associated molecular patterns (PAMPs) from diverse microorganisms in the lumen, amplify the initial immune response, and finally prime the adaptive immune system. PRRs also recognize endogenous molecules produced in stress conditions, so called DAMPs. Membrane-bound [TLRs and C-type lectin receptors (CLRs)] and cytoplasmic Nucleotide-binding oligomerization domain-like receptors or NOD-like receptors (NLRs), retinoic acid-inducible gene-I-like receptor (RLRs), absent-in-melanoma 2 (AIM2)-like receptors, and cyclic GMP-AMP synthase (cGAS) receptors act together in order to detect pathogens in multiple cellular compartments. Although TLRs are the best characterized PRRs, they are not unique in the context of IECs and IBD; others relevant receptors comprise CLRs (229) and NLRs (NOD2) (230, 231).

Focusing on the well-studied TLRs, deficiency of TLR2 is associated with aggravated colitis in DSS-treated mice (232) and multidrug resistance colitis (233). Similarly, poly(I:C)-mediated TLR3 activation protects epithelial barrier function and ameliorate DSS-induced colitis (234, 235). In contrast, several strategies based on TLR4 blocking show promising results in the context of epithelial restoration in IBD (not in the case of necrotizing enterocolitis), while constitutively activated TLR4 predisposes for DSS-colitis and colitis-induced neoplasia (236–238). Mechanistically, this is based on impaired NF-kB-mediated cytokine production and migration of epithelial cells. Although TLR5 was identified as one of the first IBD loci and its deletion triggers spontaneous colitis (239), controversial results regarding flagellin-mediated activation implies the need of future studies in this context (240). Another important candidate is TLR7, since its activation leads to production of antimicrobial peptides (AMPs) and protects against DSS (241) or TNBS colitis. Interestingly, as already mentioned in section Neuroinflammation in PD, recent studies suggest aSyn as a DAMP-activating TLRs on the surface of microglia (108, 109). This opens the hypothesis of a TLR-mediated recognition of aSyn in the gut, even via specific stimulation of intestinal epithelium or IEC subtypes. As mentioned above, it is important to consider potential specificity of TLR activation based on the conformation of the different aSyn aggregates in this context (242).

#### Intestinal Permeability in PD

PD pathogenesis is associated with “leaky gut” (Figure 1) and increased intestinal permeability (243), correlating with aSyn and LPS levels in the mucosa (190). Elevated intestinal permeability in turn promotes subsequent inflammation, and therefore, aSyn accumulation and aggregation in the ENS (192). In fact, increased expression of pro-inflammatory cytokines and glial markers, also in the gut, positively correlated with disease progression and severity. Mechanistically, recent studies have suggested that PD patients (123) and animal models of PD show altered expression and distribution of tight junction proteins, such as ZO-1, E-cadherin (244), and claudin-1 (245).

#### aSyn and Intestinal Epithelial Cells

Beyond the association between PD and decreased expression of tight junction proteins within the intestinal epithelium (123), the current knowledge about a potential interaction between aSyn and the intestinal epithelium is still scarce. The fact that EECs express aSyn make them attractive candidate players in this context [see chapter Enteroendocrine Cells (EECs)]. aSyn can be transmitted in a prion-like manner from epithelial cells to enteric neurons (30). Enteric glia is a crucial communication tool between the intestinal epithelium and the ENS. Thus, intestinal pathological conditions associated with alterations of epithelial permeability might trigger alterations of the EGCs as the declutching event for a local immune response and neuroinflammation affecting the ENS (see chapter Neuroinflammation in PD). In order to get in contact with IECs, aSyn should translocate across the mucus barrier protecting the monolayer of epithelial cells. A recent study has shown that, despite mucoadhesive properties, aSyn penetrates the mucus by inducing rearrangement of the mucin matrix (246). Other studies suggest that rather than aSyn itself, other phenomena associated with alterations of the microbiota, such as increased levels of LPS are responsible for epithelial alterations, including redistribution of ZO1 and E-cadherin (244). Enteric biofilms are produced by bacteria in the gut in order to promote their survival, and can in turn, activate local immune response, since some of their components act as DAMPs activating TLRs, for instance. Curli-containing biofilms in several experimental infection models caused alterations of the epithelial layer; the mechanism behind includes the fibrillization of aSyn (247). Undoubtedly, further research on the impact of aSyn on IECs, as well as other mechanisms explaining epithelial alterations in the context of PD pathogenesis are required.

The use of brain organoids derived from PD patient iPSCs has been extended in the last years. Recently, technical development in the field, such as co-culture of neuronal cells with astrocytes (248) and the use of assembloids have permitted modeling of cellular crosstalk between different areas of the brain (249). However, controversial opinions about the ability of these *in vitro* models to mimic complexity of the human brain still exist. In the context of PD, midbrain organoids containing dopamine-related neurons, astrocytes and oligodendrocytes (250) have demonstrated to recapitulate pathological hallmarks upon appropriate conditions (e.g., LRRK2 mutations), such as neurotoxic damage, endosomal phosphorylated aSyn, and increased mitophagy (27). Future advances regarding midbrain organoids may be the inclusion of other cell types, such as microglia, which enables to study the relevance of innate immunity in PD. Therefore, two strategies have been proposed: on one hand, the development of brain organoids including microglia (251); and on the other hand, exogenously add iPSC-derived microglia to brain organoids (252). Moreover, in order to model the BBB and the potential immune cell trafficking, neurovascular communication has been developed and implemented via organ-on-chip technology (endothelial-like cells, astrocytes, and neurons) (253).

The relevance of the gut-brain axis in PD opens the path for exploiting intestinal organoids as *in vitro* models of PD. Described in 2009, intestinal organoids or enteroids are 3D structures developed from intestinal stem cells cultures allowing the intricate differentiation of IECs, and mimicking the complex architecture of the intestinal epithelium (254). Although extremely useful in the context of of epithelial-intrinsic phenomenon, two aspects of intestinal organoids limit their use in studies dealing with microbial-epithelial communication. On one hand, the apical side of the polarized epithelium is projected toward the inside of the organoid (lumen) and makes microbial stimulation highly challenging; and on the other hand, culture conditions with high oxygen concentrations are not optimal for the growth of a vast majority of anaerobic intestinal microbiota. Moreover, some limitations also accounted in the case of co-culture settings with immune cells, for instance, the lack of nutrient support and mechanical constrains to immune cells mediated by blood flow and circulation. Thus, organ on a chip cultures mimicking the inter-organ communication and allowing the interaction with the microbiota as well, appear as suitable alternative. Highly challenging tissue engineering approaches combined with transplantation into mice have tried to implement *in vitro* systems including the ENS to co-cultures of intestinal organoids and smooth muscle cells; however, these strategies have not been successful until now, based on the lack of maturity of neuronal cells (255). More advances have been achieved in the context of immune-epithelial and microbiota-epithelial communication in organoid cultures. Addition of macrophages affected epithelial barrier function and maturity (256); while neutrophils in combination with pathological bacteria cause loss of epithelial integrity (257) and epithelial development and/or maturation is promoted by TNF-producing CD4+ T cells (258).

A step further in the field of PD research will be the combination of gut and brain organoids. Recent advances have focused on “patient-on-chip” models, such as the combination of separately developed multiorgan organoids (259); or the use of gut organ-chip models fluidically coupled to vascular endothelium lined channels (260), such as MINERVA (MIcroboita-Gut-BraiN EngineeRed platform to eVAluate intestinal microflora impact on brain functionality) (261). Experimental setups based on intestinal organoids and multiorgan organoids might provide important knowledge of the communication between the gut and the brain.

#### Enteroendocrine Cells (EECs)

Considered sensory cells within the secretory lineage of IECs, EECs represent the largest source of hormones in the body and play vital roles in many physiological processes like appetite control, sensing of gut microbiota, GI immunity, motility, barrier function, insulin and growth hormone secretion (262). Upon sensing of nutrients, EECs produce neuropeptides and hormones to the basal space. In the gut epithelium, enterochromaffin cells (ECs)—a subtype of EECs, react to mechanical forces during gut peristalsis by secreting 5-HT, accounting for 95% of body 5-HT (263). For decades, 5-HT is known as an important neurotransmitter signaling molecule, holding a key role in gut motility, secretion and pain sensation. Many studies indeed showed the link between abnormal regulation of 5-HT and GI disorders, such as IBD and irritable bowel syndrome (IBS) as well as in many CNS disorders (264, 265), suggesting a significant role of 5-HT in gut-brain-gut communication. Recently, EECs have been proposed as an alternative source for Notch ligands, supporting the stem cell population in Paneth-deficient mice (266). Therefore, it is predictable that many gut dysfunction diseases, including IBD, are associated with EECs alterations.

EECs possess a tightly organized apical brush border, and basal membrane projections (neuropods) allowing the intercellular communication with nerves and neurons (267). Interestingly, EECs show a certain overlapping expression profile with neuronal cells, such as neurotrophin receptors, pre- and post-synaptic proteins including aSyn, neurofilaments mimicking axons and their functions (neuropods), and dopamine synthesis machinery (268). Indeed, EECs not only synapse with enteric nerves (29) but also establish a direct contact with enteric glia (269). Thus, EECs can serve as a connection between the intestinal lumen and the ENS, and represents a key population in the context of gut-brain axis in neurodegenerative diseases (267). Besides direct cellular contact, EECs communicate with the ENS via the release of NT and hormones; or even act as the entry pathway for pathogens, which can then act on neurons in the gut. Most importantly, based on their neuron-like features, they can serve as niche for proteinopathy upon luminal signals, which is further supported by the expression of aSyn from these cells (30). Hence, the question arises, whether EECs may be the starting point or declutching event for aSyn pathology in the gut, which is then further transmitted to the CNS.

The fact that EECs express aSyn opens the path for the study of proteinopathy specifically in these cells. An important aspect in this context is the exposure of EECs to the lumen, which make them accessible via endoscopy, as a future early diagnostic tool of premotor PD (270). Interestingly, different TLRs (TLR1, 2, and 4) are expressed in EEC cell lines (271); while TLR4, -5, and -9 ligands induced secretion of EECs hormones in mice (272). On the other hand, *Bacteroides thetaiotaomicron* contributes to neurogenic colon activity via a TLR2- and EEC-dependent mechanism (273). Interestingly, TLR overstimulation has also been suggested in PD pathology (274). Another mechanism by which EECs contribute to the barrier function might be mediated by the expression of SCFA receptors, such as FFAR2 and FFAR3 (275–277).

Interestingly, qualitative and quantitative alterations of EECs have been associated with GI dysfunctions also observed in PD, such as constipation or alterations of transit times. Rotavirus infection courses with EEC-mediated 5-HT secretion, which activates the ENS and the extrinsic vagal afferent to the brain causing nausea, vomiting, and diarrhea (278). In contrast, increased 5-HT secretion protects intestinal barrier function due to the production of neutropic factors (279). Similar EECs-5-HT-dependent mechanisms operate also in diarrhea upon viral infections, such as Adenovirus infection (280) and even COVID-19 patients (281).

### Mechanosensations in the Gut

Mechanosensation is vital for proper function of electrically excitable organs, those constantly exposed to and/or generating mechanical forces (heart, bladder, and GI). Physiologically, all cells in the gut epithelial layer are mechanosensitive, they need to sense the static forces (e.g., stretching, crowding) to adjust cell numbers and maintain epithelial integrity. Among them, so-called mechanosensitive cells, develop specific ion channels to sense acute mechanical forces (e.g., pressure from luminal food content); these cells are important to maintain gut functions like food digestion and peristalsis. Beyond peristalsis, mechanical issues are also crucial for maintenance of epithelial architecture. It is well-known that stem cell proliferation is important to maintain tissue homeostasis and avoid pathological conditions. Interestingly, in *Drosophila*, the strict regulation of stem cells is indeed associated with food digestion via gut epithelial stretching. Changes in mechanical properties upon ingestion (gut distension), lead to the decrease of misshapen (a Hippo pathway regulator) membrane association and phosphorylation, which then stimulates stem cell activity and contributes to control intestine adaptive growth (282). During epithelial turnover, aged or damaged cells are shed into the lumen in order to leave space for newly generated cells. This process must be tightly governed to maintain epithelial integrity, and therefore requires intercellular sensing communication between shedding and neighboring cells to finally extrude the dying cell. In general, little is known about biochemical pathways governing sensing and responses to mechanical forces.

Although several membrane ion channels have been revealed as important players in this context, the recently identified Piezo channels show their notable roles in many cellular mechanosensitive processes, from light-touch sensing, controlling red blood cell volume to muscular shear stress (283). In *Drosophila* midgut, the unique Piezo isoform is expressed in low division precursor cells differentiating into EECs. Adult Piezo mutant fly showed decreased number of EECs compared to WT fly. Moreover, Piezo overexpression or increasing Ca^2+^ level in fly intestinal stem cells induced both cell proliferation and EEC differentiation (284). In zebrafish, Piezo1 ion channel is reported to participate in live cell extrusion (285) and cell division (286), in response to crowding and stretching, respectively. Disturbing cell extrusion via Piezo1 channel lead to formation of cell masses, which hypothetically can lead to tumorigenesis. Gudipaty et al. have proposed a model on how Piezo1 acts as a regulator of epithelial cell number by shifting its localization between nuclear envelope and cytoplasm/ plasma membrane in order to control cell division and extrusion (286). Altogether, these studies suggested that investigating Piezo-mediated mechanosensations will give us insights into intracellular pathways regulating cell numbers and epithelial integrity, and therefore, be relevant in the context of intestinal inflammation and tumorigenesis.

#### Peristalsis

Peristalsis, or the impulsion of food based on muscle contraction and relaxation, is regulated by sensation of mechanical forces, but the molecular mechanism behind remains elusive. Generally, peristaltic waves in small intestine consist of weak and infrequent contractions around the bolus, while they continuous and gradually increased toward the anus in the colon. Under specific circumstances, for example diarrhea, an intense and powerful peristaltic wave is triggered in the whole small intestine, which quickly relieves mucosa irritation or unusual gut distension. In the small intestine, peristalsis helps driving food against intestinal wall for nutrient absorption and persistently push it toward the large intestine. In the large intestine, peristalsis is important for feces elimination and mechanical removal of gas and bacteria. At a cellular level, when food particles are formed, EECs are stimulated to secrete 5-HT, while mechanosensory neurons in circular and longitudinal muscles are activated to declutch gut motility (287).

The muscle contraction depends on signals received from ENS or CNS, such as substance P, neuropeptide Y or inhibitory neurotransmitters including nitric oxide (NO) and vasoactive intestinal polypeptide (VIP) (288). How the excitatory and inhibitory motor neurons are activated is still a controversy, however, a population of sensory neurons in the distal colon of guinea-pig are believed to be stretch-sensitive rather than muscle tone or contraction sensitive (289).

In order to respond to mechanical stimulations, the intestinal tract contains various mechanosensitive cell types carrying membrane mechanically gated ion channels such as ECs within the epithelial layer, smooth muscles, interstitial cells of Cajal or different types of sensory neurons in the lamina propria. They sense and respond to mechanical changes in different ways; for instance, by 5-HT secretion in the case of ECs. Even though the molecular mechanism behind mechanically induced 5-HT-release in ECs is unknown, recent evidence revealed that Piezo2 ion channel is specifically expressed in human and mouse 5-HT positive ECs, and Piezo2 activation by mechanical forces is necessary for 5-HT release and mucosal secretion (290). Another study suggests that Piezo2 is selectively expressed in a large number of NeuroD1+ cells—a subset of EC cell, and mechanical stimulation of NeuroD1+ cells leads to Piezo2-dependent, but not Piezo1-dependent Ca^2+^ increase inducing 5-HT production (291). Paradoxically, a newly published study showed that 5-HT release is crucially regulated upon detection of bacterial derived single-stranded RNA by Piezo1 channel in the gut epithelium, indicative of a new potential pathway for gut and bone disorder therapies. Even though the function of the Piezo family in EECs is not clear, Piezo1 was found to regulate gut peristalsis positively *in vivo* and the lack of Piezo1 in epithelial caused whole gut transit time delay (292). Considering mentioned evidences, Piezo1 and Piezo2 channels in gut epithelium could be possible key elements to uncover the mechanism behind EECs-related mechanisms operating behind constipation and altering transit time in PD (Figure 2). This knowledge might even elucidate the phenomena explaining misfolded aSyn-EECs and reveal the initiation of PD origin.

#### Constipation and PD

The abnormal defecation and reduced peristalsis can lead to constipation. Physically, constipation occurs when there is a decrease of bowel movement frequency, due to primary (idiopathic or functional) or secondary reasons (diet or medication). Approximately 52.48% PD patients experience constipation (293), making it the most common and distressing PD gastrointestinal symptoms (Figure 1). Indeed, a study with 551.324 volunteers in Taiwan showed that participants with mild to severe constipation symptoms tended to develop PD within 5.5 years and the constipation severity correlated with the risk of having PD (294).

### Targeting the Gut for PD Treatment

Current pharmacological treatment for PD patients is based on the principle of escalating DA brain concentration, by (1) increasing/replacing DA levels; or (2) impairing its degradation. Since DA does not cross the BBB, the most commonly used drug is based on the action of Carbidopa/levodopa, a precursor of DA, which crosses the BBB and is believed to convert to DA in the brain. Other available medicines include DA agonists, monoamine oxidase type B (MAO B) inhibitors, catechol-O-methyltransferase (COMT) inhibitors, anticholinergics, Amantadine or Creatine (295). Pharmacological treatment can be also combined with surgery (deep brain stimulation) (296), gene therapy (297), immunotherapy (e.g., antibodies against aSyn) (298), or cell transplantation (299). However, none of the available therapeutic options is actually curative, nor able to stop disease progression (300, 301). Together, the need of alternative therapy strategies in PD is patent, which opens avenues for the identification of innovative strategies.

Considering gut-brain axis in the context of PD, nowadays it is suggested that PD can be, not only diagnosed based on GI manifestations, but even treated “from the gut.” This principle has been also exploited in the context of innovative strategies for levopoda therapy (302). For instance, currently used duopa therapy is based on the application of gels enabling the release of carbidopa/levodopa directly in the gut, allowing slow absorption and, therefore, impairing motion fluctuations and movement disorders. Tightly linked to intestinal function and microbiota, increasing attention has been paid to PD clinical management based on the diet, especially dietary fat. However, conflicting results do not permit drawing conclusive remarks in this context (303, 304); except for the fact that polyunsaturated fatty acid consumption has been associated with lower risk of PD (305). In accordance with the role of the microbiota in the pathogenesis of PD, several strategies modulating the microbiota demonstrated the potential in the context of PD. Antibiotics treatment ameliorate signs of PD, such as IL-1β and, TNF-α at the CNS and dopamine neuron loss (306, 307). Both pre- and pro-biotics have an effect on aSyn proteinopathy. Thus, butyrate activates aSyn autophagy and promotes barrier function of the intestinal epithelium (308). On the other hand, *Bifidobacterium* and *Lactobacillus* are able to reverse PD and PD-related constipation (309); while *Lactobacillus* promotes production of L-DOPA from L-tyrosine (310). The use of probiotics has been found to be beneficial in PD patients (311, 312) and experimental PD models (313, 314). Regarding fecal transplantation, there are controversial results; it is suggested that FMT not only improves GI symptoms (constipation) but also neuroinflammation in PD patients (315, 316); however, safety and efficacy are not clear. Experimentally, FMT lead to further decreased of *Lachnospiraceae* and *Ruminococcaceae*, and worsening of dyskinesia (191); while FMT from PD patients lead to worsening on motor symptoms in a PD model (192), but motor impairment was also observed in normal mice. FMT can impair TLR4 activation, improve gut dysbiosis, reduce activation of microglia, change NT secretion and the destruction of the substantia nigra (315). FMT can also ameliorate comorbidity in PD patients related to the GI tract, including ulcerative colitis (317).

## Animal Models of PD and GI Symptoms

As mentioned above, the interplay between neurological and GI symptoms in PD is also nicely demonstrated in animal models. Thus, here we provide a summary of currently used experimental *in vivo* models of Parkinsonism, and the occurrence of GI pathological features, as well as aSyn propagation mechanisms supported by experimental observations using corresponding models.

Classical PD models are based on toxin-induced motor manifestations. Intragastric injection of rotenone causes Parkinsonism in mice, without increased systemic rotenone levels (133). The presence of aSyn in the GI tract or ENS depends on factors such as the administration route, dose or length of exposure. Thus, chronic exposure of rotenone involves non-motor GI symptoms (318), however, did not delay gastric emptying (319). Interestingly, rotenone toxicity is associated with changes in the microbiota composition, such as decreased *Bifidobacterium* and increased *Rikenellaceae* or *Allobaculum* (320); while severity of the symptoms is associated with decreased *Lactobacillus* and increased reactivity to LPS (245). In addition, cell toxicity is induced by the prodrug MPTP and the neurotoxin MPP+, causing dopaminergic neurons/tyrosine hydroxylase + (TH) neuron destruction in the brain and in the colonic ENS (321, 322). However, controversial data exists about the outcome of this GI affectation, and it is not clear if this is associated with increased intestinal motility (323) or constipation (322, 324). Strikingly, recent publications support a MPTP-mediated intestinal immune response, which might be provoked by activation of monocytes (325). Furthermore, direct brain injection of 6-OHDA induces PD-like constipation, delayed gastric emptying, and enteric inflammation (326, 327). Last but not least, paraquat injection into rats also demonstrated the relevance of the gut-brain axis, since it evokes reduced gastric motility tone and increased aSyn immunoreactivity in the DMV, which is blocked upon vagotomy (328, 329).

Most experimental genetic models are based on the induced expression of aSyn or mutations on the gene encoding for aSyn. These models recapitulate aSyn aggregation, similar to PD patients, however, require more time for pathological manifestations. In accordance with the gut-brain axis hypothesis, these models confirm that GI dysfunction and non-motor symptoms might represent early pathological features. The most commonly used model is the Thy1-aSyn overexpression model, which presents GI manifestations, delayed colon transit time and defecation accompanied by aSyn accumulation in colonic myenteric plexus (330, 331). CNS pathology in Thy1-aSyn mice is reduced upon microbial depletion, while FMT from PD causes worsening of the phenotype (192). However, a recent study claims that levels of LPS rather than microbiota alterations in Thy1-aSyn mice are responsible for colon intestinal permeability dysfunction and early motor manifestations (244). Other genetic models taking advantage of mutations on the aSyn gene, show aSyn accumulation in the olfactory bulb, myenteric plexus and adrenal neurons (aSyn-A53T) or accumulation of phospho-aSyn, slower transit time, abnormal stool and neuroinflammation at the ENS (PrP-A53T-aSyn) (332, 333). Even unique GI affectations, without motor dysfunction can be observed (BAC-A53T-aSyn) (334).

Mutations in *PINK1* and *PARK2* are associated with PD and activation of immune responses via modulation of mitophagy/autophagy (335). Interestingly, immune response in the context of *PINK1* knockout mice is regulated by the microbiota, since colonization with bacteria leads to T cell mediated destruction of dopaminergic neurons in the periphery and the brain (336). On the other hand, the MitoPark model represents a noticeable example of experimental recapitulation of GI dysfunction and dysbiosis in PD Non-motor symptoms in this model include decreased motility and gradual progression of colon transit times, reduced fecal water content and activation of glial cells in the myenteric plexus. Disease progression in this model goes along with loss of TH+ neurons, reduction of central and intestinal DA levels, as well as changes in the microbiota composition (337).

As mentioned above, another important aspect within the gut-brain axis concept is the propagation route for aSyn. Thus, researchers in the field have concentrated on the development of experimental models based on the injection of aSyn. Therefore, pathological aSyn can be isolated from post-mortem human tissue; or recombinant aSyn preformed fibrils (PFF) are experimentally prepared. It has been demonstrated that the injection of patient-derived pathological aSyn directly into the gut leads to deposition of aSyn in myenteric neurons and intestinal inflammation in A53T transgenic mice (338). Intragastric aSyn can be transmitted to the brain in rats (142). Moreover, the injection of recombinant PFF in the olfactory bulb in WT mice caused the spread of aSyn to distant areas of the brain (339). While spreading of aSyn occurred only in aSyn transgenic mice upon injection into gastric wall and not in WT mice (340). Inoculation of PFFs in the duodenum of mice led to GI deficits and physiological changes of the ENS in addition to changes of aSyn histopathology in the midbrain and subsequent motor defects in elder, but not in young mice (28).

## Conclusion

As outlined in our review, the disease mechanisms of PD are complex and exhibit a variety of pathological facets. GI manifestations are the most significant symptoms in the prodromal phase of PD (115), suggesting the direct communication of gut and brain. Recent studies have shown that pathogenic aSyn found within the GI system are able to spread and reach the CNS (28, 142, 339). In addition, the role of constipation in PD seems to support the hypothesis that the pathological pathway of PD spreads from the intestine to the brain. Besides, EECs were found to express aSyn and link directly to aSyn-containing nerves, creating neural circuit between the gut and nervous system. This raised an interesting hypothesis that the root of PD might start from misfolded aSyn in EECs, which is transmitted to the nervous system (30). Moreover, constipation is the most troublesome PD-gastrointestinal symptom and likely regulated by abnormal gut peristalsis (293). Accordingly, investigating the roles of EEC-mechanosensitive ion channels, which indeed was proven to be associated to peristalsis, could explain the reason why aSyn in ECs is misfolded, and reveal the mechanism behind PD origin.

Correspondingly, gut inflammation is a main pathological feature occurring in PD and IBD. Inflammatory processes and aSyn pathology appear to be extraordinarily linked to each other. In connection with inflammation, aSyn and its aggregated forms seem to mediate inflammatory responses by TLR activation (108, 109). This indicates the possibility of TLR-mediated release of proinflammatory cytokines in the gut by specific stimulation of IEC. Furthermore, IECs appear as key factor in inflammatory response, as they create a protective barrier against luminal antigens and microbes, helping to preserve gut homeostasis. IEC alterations, for example cytoskeletal rearrangement (12) or cell-to-cell adherens junction reorganization (341) could disturb the epithelial integrity and lead to intestinal permeability as seen in CD patients (342). In addition, PD pathogenesis is also associated with an increased intestinal permeability (243) along with impaired BBB function (97), promoting bidirectional inflammation cascades between the gut and the brain.

Many different routes for transmission between neuronal networks and intestinal cells are described to propagate aSyn pathology. Of interest, extracellular exosomes found in blood and CSF of PD patients have been described to spread pathology (151). Moreover, the vagus nerve is considered to be the most important bidirectional connection between these two organs (141). However, within this context, clinical studies investigating the origin of PD progression are still elusive.

Lastly, it is interesting that dysbiosis is a common feature in PD and IBD (168, 169). In order to affect ENS-specific pathways and spreading to the CNS, a connection between the GI lumen and the neurons/enteroglia is necessary. The intestinal epithelium is in direct contact with luminal content and therefore, acts as a physical and immunological barrier in the gut. Hence, a disturbance of the intestinal sealing allows direct contact of pathological factors and cells of the ENS. Interestingly, IBD (221) as well as PD (243) patients can suffer from intestinal inflammation concomitantly exhibiting a leaky gut. The disturbance of intestinal barrier function has been suggested to promote aSyn aggregation in the ENS, which is further able to spread to the CNS (30, 304), along the so-called gut-brain axis.

In recent years, numerous studies have been addressing the role of the gut-brain axis in neurodegenerative disorders, like PD. However, there are still open questions regarding the understanding about its impact in disease progression and regulation. Further studies and comparisons of disease mechanisms of PD and IBD, as presented in this review, might help to connect missing dots and shed light into the role of aSyn aggregation within the intestine as well as intestinal inflammation in PD. A detailed comprehension of the mechanisms and regulation of the gut-brain axis is essential to establish novel disease biomarkers, clinical read-outs and identify novel targets for (early) treatment strategies.

## Author Contributions

AD, PN, FZ, and RL-P wrote the manuscript. MN read the manuscript and contributed to the finalized version. All authors approved the final version of the manuscript.

## Conflict of Interest

The authors declare that the research was conducted in the absence of any commercial or financial relationships that could be construed as a potential conflict of interest.

## Glossary

6-OHDA, 6-Hydroxydopamine; AIEC, Adherent invasive Escherichia coli; AMPs, Antimicrobial peptides; aSyn, alpha-Synuclein; BBB, Blood-brain barrier; BDNF, Brain-derived neurotrophic factor; CD, Crohn's disease; cGAS, cyclic GMP-AMP synthase; CLR, C-type lectin receptor; CNS, Central nervous system; COMT, Catechol-O-methyltransferase; CSF, Cerebrospinal fluid; CXCL, Chemokine C-X-X- motif ligand; DA, Dopamine; DAMP, Damage-associated molecular pattern; DLB, Dementia with Lewy Bodies; DMV, Dorsal motor nucleus of the vagus nerve; DSS, Dextran sodium sulfate; EC, Enterochromaffin cells; EEC, Enteroendocrine cells; EGC, Enteroglial cells; EGF, Epidermal growth factor; ENS, Enteric nervous system; FFAR, Free fatty acid receptor; FMT, Fecal Microbiota Transplantation; GABA, γ-Amicobutyric acid; GDNF, Glial cell-line derived neurotrophic factor; GFAP, Glial fibrillary acidic protein; GI, gastrointestinal; GMP-AMP, Guanosin monophosphate-adenosine monophosphate; GPCRS, G-Protein coupled receptors; GWAS, Genome-Wide-Association studies; HSV1, Herpes simplex virus type 1; IBD, Inflammatory bowel disease; IEC, Intestinal epithelial cells; IF, Interferon; IL, Interleukin; iPSC, Induced pluripotent stem cells; LB, Lewy Bodies; L-DOPA, L-3,4-dihydroxy-L-phenylalanine; LN, Lewy neuritis; LRRK2, Leucin-rich repeat kinase 2; LP, Lamina propria; LPS, Lipopolysaccharide; MAO-B, Monoamine oxidase type B; MINERVA, MIcroboita-gut-braiN EngineeRed platform to eVAluate intestinal microflora impact on brain functionality; MPP, 1-Methyl-4-phenylpyridinium; MPTP, 1-Methyl-4-phenyl-1,2,3,6-tetrahydropyridin; MSA, Multiple system atrophy; MWAS, Metagenome-wide association studies; NAC, Non-amyloid-β component; NF-kB, Nuclear factor “kappa-light-chain-enhancer” of activated B-cells; NLR, NOD-like receptor; NO, Nitric oxide; NOD, Nucleotide-binding oligomerization domain; NT, Neurotransmitter; PAMPs, Pathogen-associated molecular patterns; PD, Parkinson's disease; PFF, Preformed fibrils; PNS, Peripheral nervous system; PRRs, Pattern-recognition receptors; PSD-95, postsynaptic density protein 95; RLR, Retinoic acid-inducible gene-like receptor; SCFA, Short-chain fatty acids; SNpc, Substantia nigra pars compacta; TGFα, Transforming growth factor α; TH, Tyrosine hydroxylase; Th, T helper cells; TLR, Toll-like receptor; TNBS, Trinitrobenzene sulfate; TNF, Tumor necrosis factor; UC, Ulcerative colitis; VIP, Vasoactive intestinal polypeptide; ZO, Zonula occludens.
